# Polyglutamine variation in a flowering time protein correlates with island age in a Hawaiian plant radiation

**DOI:** 10.1186/1471-2148-7-105

**Published:** 2007-07-02

**Authors:** Charlotte Lindqvist, Liisa Laakkonen, Victor A Albert

**Affiliations:** 1 Natural History Museum, University of Oslo, P.O. Box 1172 Blindern, 0318 Oslo, Norway; 2 Helsinki Bioenergetics Group, Programme for Structural Biology and Biophysics, Institute of Biotechnology, University of Helsinki, Biocenter 3 (Viikinkaari 1), PB 65, FIN-00014, Helsinki, Finland

## Abstract

**Background:**

A controversial topic in evolutionary developmental biology is whether morphological diversification in natural populations can be driven by expansions and contractions of amino acid repeats in proteins. To promote adaptation, selection on protein length variation must overcome deleterious effects of multiple correlated traits (pleiotropy). Thus far, systems that demonstrate this capacity include only ancient or artificial morphological diversifications. The Hawaiian Islands, with their linear geological sequence, present a unique environment to study recent, natural radiations. We have focused our research on the Hawaiian endemic mints (Lamiaceae), a large and diverse lineage with paradoxically low genetic variation, in order to test whether a direct relationship between coding-sequence repeat diversity and morphological change can be observed in an actively evolving system.

**Results:**

Here we show that in the Hawaiian mints, extensive polyglutamine (CAG codon repeat) polymorphism within a homolog of the pleiotropic flowering time protein and abscisic acid receptor FCA tracks the natural environmental cline of the island chain, consequent with island age, across a period of 5 million years. CAG expansions, perhaps following their natural tendency to elongate, are more frequent in colonists of recently-formed, nutrient-rich islands than in their forebears on older, nutrient-poor islands. Values for several quantitative morphological variables related to reproductive investment, known from Arabidopsis *fca *mutant studies, weakly though positively correlate with increasing glutamine tract length. Together with protein modeling of FCA, which indicates that longer polyglutamine tracts could induce suboptimally mobile functional domains, we suggest that CAG expansions may form slightly deleterious alleles (with respect to protein function) that become fixed in founder populations.

**Conclusion:**

In the Hawaiian mint *FCA *system, we infer that contraction of slightly deleterious CAG repeats occurred because of competition for resources along the natural environmental cline of the island chain. The observed geographical structure of *FCA *variation and its correlation with morphologies expected from Arabidopsis mutant studies may indicate that developmental pleiotropy played a role in the diversification of the mints. This discovery is important in that it concurs with other suggestions that repetitive amino acid motifs might provide a mechanism for driving morphological evolution, and that variation at such motifs might permit rapid tuning to environmental change.

## Background

The genetic mechanisms underlying organismal radiations are of great interest to biologists. Whereas genetic redundancy, differential regulation of gene transcription, and alternative RNA splicing to produce protein variants have each been implicated as fundamental means by which evolution has tinkered with morphology, less importance has been demonstrated for specific amino acid (AA) substitutions in coding regions [1-5]. A prominent reason for this difference is that the first three mechanisms can better escape deleterious effects caused by pleiotropy (the covariation of phenotypic traits) [1]. Still, AA motifs of varying lengths in pleiotropic proteins [6,7] have been correlated with morphological radiations, but only at the level of entire subphyla (deep time) [8,9] or among artificially selected dog breeds (historical time) [10,11].

Here we present a case in which shifting length of a polyglutamine (polyQ) tract in a highly pleiotropic protein may contribute to morphological radiation and incipient speciation along a natural geological gradient of Pliocene to modern age. The Hawaiian Islands are an isolated volcanic archipelago formed by plate movement over a mantle plume, with the consequence that islands evolve and subside in a linear geographic manner [12]. The three genera and ca. 60 species of endemic mints (Lamiaceae) represent one of the largest Hawaiian plant radiations. They originated from polyploid (likely octoploid) North American ancestors and diversified from a single introduction to the Hawaiian Islands [13,14]. Their morphological and ecological variation is extensive; plants range from subalpine vines to rainforest shrubs, flowers may have either bird or insect pollinated anatomies, and seed dispersal patterns may depend on either dry or fleshy fruits [15]. In contrast to this extensive diversity, however, genetic variation in nuclear and chloroplast DNA sequence markers has been found to be remarkably low, resulting in a lack of phylogenetic resolution among representatives of the two largest genera, *Phyllostegia *and *Stenogyne *[13].

We isolated a *FCA *homolog from an expressed sequence tag (EST) library for the Hawaiian endemic mint *Stenogyne rugosa *(Lamiaceae) [16]. The FCA protein of Arabidopsis, originally isolated as a flowering time gene [17], is a receptor for the plant hormone abscisic acid (ABA) [18]. Although highly pleiotropic [19], *FCA *is nonetheless finely autoregulated [20] such that flowering time is modulated in different mutant alleles [19,21]. Phenotypic features of *fca *mutant plants, likely linked to late flowering, include increased leaf number, leaf area, and size of petals, stamens, carpels and fruits [19]. An additional phenotype is reduction of the secondary root system [18,22].

In the rice FCA homolog a 9-residue polyQ tract occurs directly C-terminal to its WW protein-protein interaction domain [23], which in Arabidopsis is crucial for proper self-processing of *FCA *pre-mRNA [20]. We document that the Hawaiian endemic mints show considerable length variation at the same glutamine repeat and that this variation is temporally coincident with the emergence and subsidence of islands in the Hawaiian chain. Since polymorphic polyQ tracts in mammalian proteins are known to be responsible for a number of human neurological disorders at critical lengths [24], we considered the possibility that the mint FCA-like repeat motif could also have phenotypic consequences. Here, we describe how FCA-like polyQ variation and its island-wise distribution may have contributed to the rapid morphological diversification of the Hawaiian mints.

## Results and Discussion

### The polyglutamine tract in FCA-like proteins varies in length

**Figure 1:**
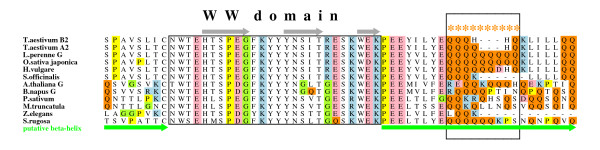
**FCA homolog sequence alignment around the WW domain**. The WW domain is shown boxed, with probable secondary structure marked (arrows for beta strands). The hypothesized beta-helix between the second RNA recognition motif (RRM) and the WW domain, and extending past the WW, is marked with a thick green line under the alignment. The polyglutamine region explored here is shown boxed, and with asterisks above amino acid residues. Sequences shown in the alignment: *T. aestivum *(*Triticum*), AAP84419 and AAP84418; *L. perenne *(*Lolium*), AAT72460; *O. sativa *(*Oryza*), AAW62371; *H. vulgare *(*Hordeum*), AAF97846; *S. officinarum *(*Saccharum*), CA085029; *A. thaliana *(*Arabidopsis*), AAW38964; *B. napus *(*Brassica*), AAL61622; *P. sativum *(*Pisum*), AAX20016; *M. truncatula *(*Medicago*), ABE82791; *Z. elegans *(*Zinnia*), AU291241; and *S. rugosa *(*Stenogyne*), EU005232. See Additional file 1 for the complete protein alignment and further explanation of structural features.

Before extensive experimentation with the mints, we investigated the organismic distribution of the polyQ tract by surveying databases for FCA homologs. We found FCA-like proteins only in land plants, but these extended in phylogenetic depth to mosses, which diverged from seed plants over 400 My (million years) ago [25]. Substantial variation in the polyQ repeat was obvious among species, and multiple *Triticum *FCA-like proteins in the database demonstrated polyQ polymorphism (Figure 1; see also Additional file 1).

### Extensive polyglutamine polymorphism among the Hawaiian mints

**Figure 2:**
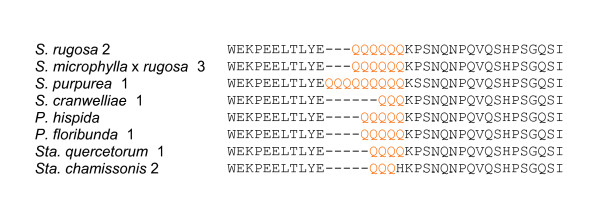
**A partial FCA protein sequence alignment of selected mint taxa**. The polyQ stretch (orange) is directly C-terminal of the WW domain. *Stenogyne cranwelliae *1 and *Phyllostegia hispida *are homozygotes, confirming the base pair/Q-tract calibration.

We genotyped 92 different Hawaiian mint individuals (representing 44 species and five presumed hybrid taxa) for polyQ variation (Additional file 2). A total number of 19 different alleles were discovered, with multiple alleles in some individuals (up to 11 in one presumed hexadecaploid individual, with an average of 2.9 alleles per individual), and an allele size range of 81–135 base pairs (bp) (see Additional file 2). Direct sequencing of selected individuals (including homozygotes) confirmed the repeat pattern, with an allele size of 87 bp corresponding to one CAG repeat, and therefore a range of 0–17 Q residues and one AA deletion at 81 bp (Figure 2).

For further analysis of the observed variation, we pooled alleles across the current taxonomy [15] since (i) sequence-based evidence for intergeneric hybridization, (ii) amplified fragment length polymorphism (AFLP) data indicating interspecific gene flow [13], and (iii) considerable non-hierarchical EST-SSR diversity [16,26] together suggest that the Hawaiian mints may best be considered a metapopulation expressing only emergent macroevolutionary patterns, with demes identified as morphospecies by taxonomists [13].

### Selection occurs along a geological gradient

**Figure 3:**
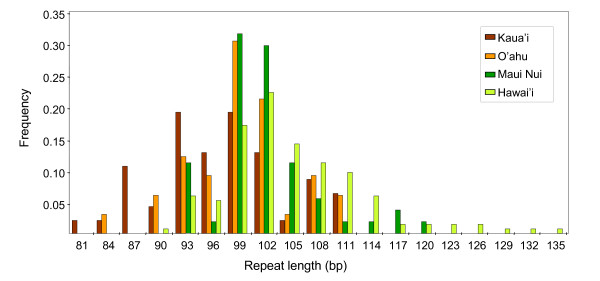
**Island-island frequency distributions of *FCA*-like alleles among the Hawaiian mints**. Note the right → left shift in frequency distributions as islands age, from Hawai'i to Kaua'i. All alleles from each individual were pooled by island. bp, base pairs.

**Figure 4:**
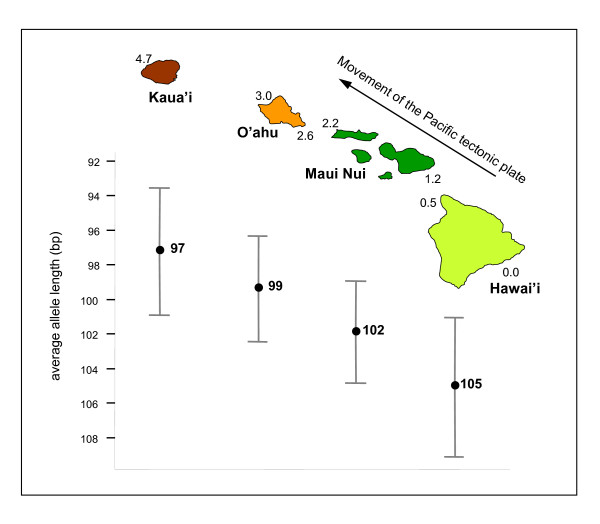
**Average allele lengths of *FCA *homologs shift with island age in the Hawaiian chain**. Longer alleles are more frequent on younger islands. The mean differences are statistically significant (Kruskal-Wallis and ANOVA, *P *< 0.001). See Table 1 for island-island Tamhane's tests. X-axis, island age (in millions of years, decreasing, as indicated next to representative volcanoes [12]). Whiskers indicate ± 0.5 standard deviations around the means.

The overall distribution of *FCA*-like allele frequencies resembles a normal distribution around an optimum of 99–102 bp (5–6 repeats), but in particular, its right tail where polyQ repeats are longest renders the distribution non-normal (Kolmogorov-Smirnov, *P *< 0.001). When our data are grouped by island into (i) Hawai'i, (ii) the Maui Nui Complex (which includes the present-day islands Kaho'olawe, Lana'i, Maui, and Moloka'i [12]), (iii) O'ahu and (iv) Kaua'i, the allele frequency distributions shift to the left with increasing island age (Figure 3). This pattern is further demonstrated by repeat length variation being significantly different (Kruskal-Wallis or one-way ANOVA, *P *< 0.001) on an island-to-island basis, with shorter repeats found on older islands, and longer ones on younger islands (Figure 4). Post hoc Tamhane's T2 tests revealed stepwise significant differences that reflect island age (Table 1).

**Table 1:** Post hoc Tamhane's T2 Test of allele length means among four Hawaiian islands. Maui Nui represents a single island complex now separated into Kaho'olawe, Lana'i, Maui, and Moloka'i. Significant *P *values (*P *< 0.05) are shown in bold.

**Group (I)**	**Group (J)**	**Mean diff. (I-J)**	**Std. error**	***P***
Kaua'i	O'ahu	-2.151	1.511	0.646
	Maui Nui	-4.676	1.343	**0.005**
	Hawai'i	-7.832	1.283	**0.000**
O'ahu	Kaua'i	2.151	1.511	0.646
	Maui Nui	-2.525	1.327	0.317
	Hawai'i	-5.681	1.267	**0.000**
Maui Nui	Kaua'i	4.676	1.343	**0.005**
	O'ahu	2.525	1.327	0.317
	Hawai'i	-3.156	1.061	**0.021**
Hawai'i	Kaua'i	7.832	1.283	**0.000**
	O'ahu	5.681	1.267	**0.000**
	Maui Nui	3.156	1.061	**0.021**

CAG repeats are known to be prone to elongation via replication slippage [27]. Theoretical and empirical work on non-CAG SSRs has suggested that expansion rates for particular repeat loci are basically constant, whereas contraction rates appear to be exponential [28]. Under this model, at a certain critical repeat length the two rates should be equal and repeat allele frequencies should be normal at equilibrium. However, this critical value, and the allele equilibrium, can of course shift under selection. Since mint populations on younger islands (longer repeats; Figure 4) likely descend from those present already on older ones (shorter repeats, Figure 4) [12], under a constant rate of CAG expansion our data are consistent with critical allele lengths decreasing with island age as islands form and subside and selection pressures increase.

**Figure 5:**
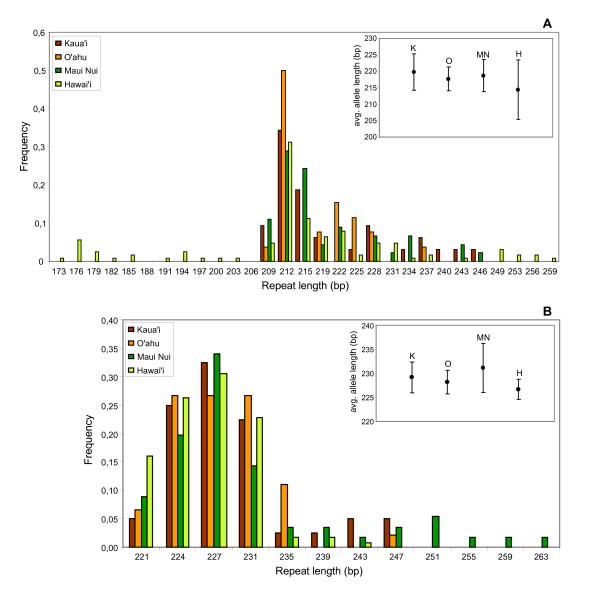
**Frequency distributions of SSR alleles for two additional loci**. Unlike the Hawaiian mints' *FCA*-like locus, frequency distributions of SSR alleles for two additional loci do not show archipelago-wide geographic progression. A, unigene 260708 (no annotation); B, unigene 261064 (annotated as At4g23400.1-major intrinsic family protein/MIP family protein [16]). Insets, average allele lengths for each island with ± 0.5 standard deviations. As described previously [16], the frequency distribution for A shows both left and right tails, representing samples principally from the island of Hawai'i. In B, the allele frequency distribution is substantially right-shifted, the four longest alleles representing a single taxon from Maui Nui (*Stenogyne bifida*). Numbers of individuals genotyped for A and B, respectively, were 93 and 91. A, Kruskal-Wallis and ANOVA n.s.; B, Kruskal-Wallis *P *< 0.05, ANOVA *P *< 0.001. B, Tamhane's T2 is significant only for the Maui Nui/Hawai'i post hoc comparison, *P *< 0.05.

To bolster the case that the observed allelic distributions are specific to *FCA*-like genes, we genotyped equally large sets of Hawaiian mint individuals for several other EST-SSR loci [16], and the *FCA *homolog was the only locus that displayed a clear geographic repeat progression (two such counterexamples are shown in Figure 5) [26]. To investigate further, we also genotyped a large number of individuals (*N *= 53) of the Hawaiian mints' parent lineage within the genus *Stachys*, which have stable, continental distributions [14]. This group followed the expected pattern, with an average inferred repeat length (4 repeats) slightly lower than that for Kaua'i (4.33 repeats).

### Morphological variables correlate with glutamine repeat lengths

**Figure 6:**
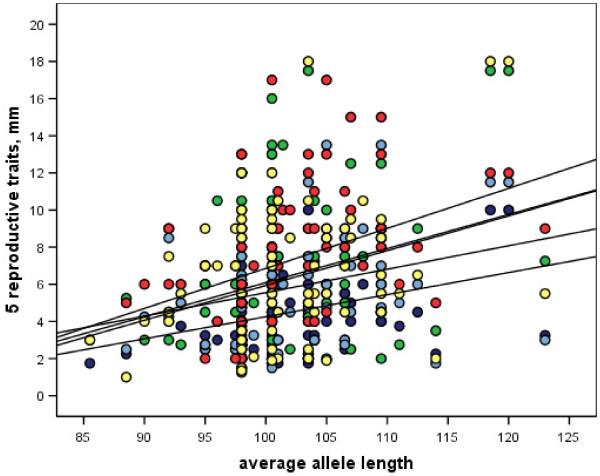
**Linear regressions of five reproductive morphological traits against *FCA *homolog average allele lengths**. Five reproductive morphological traits show similar linear correlations with *FCA *homolog average allele length per Hawaiian mint individual. The x-axis is average allele length, and the y axis represents measurements in millimeters. Dark blue = nutlet size; green = corolla lower lip length, light blue = corolla upper lip length, red = number of flowers per verticillaster, yellow = pedicel length. Regression lines, from top to bottom at the y-intercept,: pedicel length, flowers per verticillaster, corolla upper lip length, corolla lower lip length, nutlet size. See Table 2 for *R*^2 ^and significance values. Note that none of these morphological variables show significant correlation with average allele lengths for the other two loci shown in Figure 5.

To examine the possible involvement of the polyQ repeat in the pleiotropic function of the FCA-like protein, we analyzed correlations between repeat length and measures for selected morphological variables [15]. We used average repeat lengths as placeholders for genotypes. We found significant positive associations (using linear regression) of *FCA*-like allele length with several features related to reproductive investment (Table 2). Although *R*^2 ^values were relatively low, as might be expected from subtle developmental influence, slopes were similar: allele length always increased with values of quantitative morphological variables (Figure 6). Importantly, none of these traits showed significant island-wise partitioning (Kruskal-Wallis or one-way ANOVA, *P *> 0.05), which suggests genotype-phenotype correlation independent from geography. Furthermore, none of the reproductive features showed significant correlation with allele lengths of the other loci figured in Figure 5[26]. Taken together, these results support the hypothesis that the observed phenotypes may be linked to *FCA*-like genotypes rather than to underlying population structuring. It follows from this hypothesis that longer and longer *FCA*-like alleles may be equivalent to Arabidopsis *fca *mutants of increasing severity [21], for which later flowering times would be expected to increase reproductive investment [19]. However, Hawaiian mints are perennials, unlike annual Arabidopsis, so vegetative-reproductive intervals will require detailed study to assess correlation with FCA polyQ length variation.

**Table 2:** Linear regression estimations between allele length means and average values of morphological measurements. Allele length means (independent variable) and average values of morphological measurements (dependent variable) for the following different groups of data points were subjected to linear regression analysis: all Hawaiian mint accessions, *Phyllostegia *accessions only, *Stenogyne *accessions only, and accessions representing the four different island groups, Hawai'i, Maui Nui, O'ahu, and Kaua'i. Maui Nui represents a single island complex now separated into Kaho'olawe, Lana'i, Maui, and Moloka'i. Significant *P *values (*P *< 0.05) are shown in bold. No adjustments for multiple tests were made (see Methods).

**Dependent variable (avg.)**	**Data points evaluated**	***N***	**R Square**	**Constant**	**b1**	***P***
Nutlet size	all mints	84	0.114	-7.631	0.119	**0.002**
	*Phyllostegia*	44	0.163	-8.599	0.123	**0.007**
	*Stenogyne*	39	0.071	-6.436	0.113	0.101
	Hawai'i	38	0.067	-2.826	0.072	0.116
	Maui Nui	22	0.076	-7.873	0.117	0.215
	O'ahu	12	0.472	-25.315	0.299	**0.014**
	Kaua'i	12	0.182	-14.158	0.192	0.167
Corolla lower lip length	all mints	84	0.075	-12.792	0.187	**0.011**
	*Phyllostegia*	42	0.116	-11.812	0.200	**0.027**
	*Stenogyne*	41	0.028	-3.235	0.068	0.296
	Hawai'i	38	0.122	-20.890	0.260	**0.032**
	Maui Nui	22	0.240	-33.907	0.387	**0.021**
	O'ahu	12	0.018	-1.197	0.085	0.679
	Kaua'i	12	0.032	-1.871	0.093	0.581
Corolla upper lip length	all mints	85	0.073	-7.066	0.126	**0.013**
	*Phyllostegia*	43	0.174	-11.960	0.161	**0.005**
	*Stenogyne*	41	0.084	-6.907	0.138	0.066
	Hawai'i	38	0.033	-1.027	0.070	0.278
	Maui Nui	22	0.000	4.831	0.007	0.961
	O'ahu	12	0.476	-44.477	0.504	**0.013**
	Kaua'i	13	0.110	-7.375	0.129	0.269
Number of flowers per verticillaster	all mints	87	0.149	-14.690	0.215	**0.000**
	*Phyllostegia*	44	0.199	-15.368	0.228	**0.002**
	*Stenogyne*	42	0.064	-8.781	0.152	0.106
	Hawai'i	38	0.127	-16.148	0.235	**0.028**
	Maui Nui	22	0.150	-20.458	0.270	0.075
	O'ahu	12	0.017	11.456	-0.051	0.683
	Kaua'i	15	0.188	-6.556	0.124	0.107
Corolla tube length	all mints	86	0.005	6.368	0.082	0.518
	*Phyllostegia*	44	0.013	5.794	0.051	0.464
	*Stenogyne*	41	0.030	-10.470	0.287	0.279
	Hawai'i	38	0.009	23.323	-0.080	0.563
	Maui Nui	22	0.062	67.070	-0.510	0.264
	O'ahu	12	0.520	-103.578	1.200	**0.008**
	Kaua'i	14	0.281	-13.663	0.271	0.051
Corolla tube length^a^	all mints	84	0.022	0.636	0.131	0.178
excl. *S. kamehamehae*	*Phyllostegia*	44	0.013	5.794	0.051	0.464
	*Stenogyne*	39	0.131	-24.717	0.413	**0.024**
	Hawai'i	38	0.009	23.323	-0.080	0.563
	Maui Nui	20	0.055	31.265	-0.189	0.322
	O'ahu	12	0.520	-103.578	1.200	**0.008**
	Kaua'i	14	0.281	-13.663	0.271	0.051
Corolla size	all mints	84	0.041	-2577.214	32.336	0.065
(upper·lower·tube)	*Phyllostegia*	42	0.126	-3335.353	38.880	**0.021**
	*Stenogyne*	41	0.016	-2130.259	28.476	0.433
	Hawai'i	38	0.056	-2146.946	27.223	0.153
	Maui Nui	22	0.003	-742.692	15.059	0.811
	O'ahu	12	0.422	-12105.969	131.029	**0.022**
	Kaua'i	12	0.258	-1528.194	20.864	0.092
Corolla size^a^	all mints	82	0.117	-3499.719	40.347	**0.002**
(upper·lower·tube)	*Phyllostegia*	42	0.126	-3335.353	38.880	**0.021**
excl.*S. kamehamehae*	*Stenogyne*	39	0.140	-4311.797	47.821	**0.019**
	Hawai'i	38	0.056	-2146.946	27.223	0.153
	Maui Nui	20	0.304	-5915.636	61.546	**0.012**
	O'ahu	12	0.422	-12105.969	131.029	**0.022**
	Kaua'i	12	0.258	-1528.194	20.864	0.092
Pedicel length	all mints	87	0.042	-10.461	0.172	0.057
	*Phyllostegia*	44	0.035	-10.391	0.178	0.223
	*Stenogyne*	42	0.056	-5.625	0.118	0.132
	Hawai'i	38	0.004	0.946	0.071	0.702
	Maui Nui	22	0.121	-22.769	0.279	0.113
	O'ahu	12	0.336	-20.582	0.277	**0.048**
	Kaua'i	15	0.042	-2.592	0.084	0.464
Pedicel length^a^	all mints	85	0.106	-12.258	0.183	**0.002**
excl. *P. warshaueri*	*Phyllostegia*	42	0.125	-14.630	0.208	**0.022**
	*Stenogyne*	42	0.056	-5.625	0.118	0.132
	Hawai'i	36	0.089	-11.678	0.180	0.078
	Maui Nui	22	0.121	-22.769	0.279	0.113
	O'ahu	12	0.336	-20.582	0.277	**0.048**
	Kaua'i	15	0.042	-2.592	0.084	0.464
Calyx length	all mints	87	0.004	4.339	0.041	0.538
	*Phyllostegia*	44	0.082	-5.538	0.114	0.060
	*Stenogyne*	42	0.000	11.554	-0.005	0.961
	Hawai'i	38	0.002	6.140	0.026	0.771
	Maui Nui	22	0.085	35.650	-0.269	0.189
	O'ahu	12	0.451	-48.521	0.572	**0.017**
	Kaua'i	15	0.004	5.393	0.031	0.826
Leaf area	all mints	87	0.002	87.498	-0.347	0.713
(length*width)	*Phyllostegia*	44	0.021	210.761	-1.242	0.343
	*Stenogyne*	42	0.015	-5.910	0.246	0.435
	Hawai'i	38	0.017	-48.992	0.857	0.431
	Maui Nui	22	0.058	-137.727	1.754	0.280
	O'ahu	12	0.004	206.664	-1.083	0.852
	Kaua'i	15	0.000	69.246	-0.079	0.961

^a^For some corolla tube length regressions, data points for *S. kamehamehae *were excluded since they were extreme outliers. Likewise, some curve estimations for pedicel length also excluded outlier data points for *P. warshaueri*.

We also investigated the possible influence of taxonomic effects using partitioned regression analyses. These experiments were meant to control for any lineage-based effects that could reflect underlying (yet undiscovered) population structuring. Indeed, for six of seven morphological variables, data based on *Phyllostegia *alone showed significance, but for one of these seven traits, both *Stenogyne *and *Phyllostegia *data points produced significant regression lines. In every case, *R*^2 ^increased in each taxonomically partitioned analysis (Table 2). Clearly then, morphospecies assigned to *Phyllostegia *provide most of the allelic correlation in our pooled analysis of Hawaiian mints. Nevertheless, taxonomic partitioning among the *FCA*-like alleles alone could be excluded, since repeat lengths were not significantly different between the genera *Phyllostegia *and *Stenogyne *(Mann-Whitney U, *P *= 0.206). Our interpretation is that the FCA-like protein may be only one factor regulating polygenic trait differences underlying morphological distinction between the currently recognized genera.

We also performed island-wise regression analyses, the results of which (excluding spurious significance for O'ahu, which has marginal sample size *N *= 12) demonstrated that three reproductive morphological variables significantly correlated with *FCA*-like allele length on younger islands only (Table 2). These findings echo the right-hand tail on the *FCA *homolog allele distributions (Figure 3).

### Selection in the context of the Hawaiian environment

An easily understood whole-island selective force that may be operating to reduce CAG repeat length over time is the well-known nutrient cline of the Hawaiian Island chain. Phosphorus (P), in particular, leaches from volcanic soils as they age, generating a competitive environment for plant growth [29]. Another growth-compromising factor that may have influenced present-day older islands is periodic drying during glacial periods [30]. Although we were not able to directly observe it, competition may be manifested at the level of the *fca *root phenotype (reduced secondary root systems [18,22]) since it has been shown that availability of P can have a dramatic effect on root dynamics in the Hawaiian Islands. Sites low in P on Kaua'i show greater living fine-root mass and root length density than do younger sites on Hawai'i [31]. Although it remains to be empirically demonstrated, our evidence is consistent with *FCA*-like alleles of reduced wild-type function permitting greater allocation of resources to reproduction on younger, nutrient-rich islands where benefits of extensive root systems are less important. It is even possible that positive selection on slightly deleterious alleles could occur if reproductive isolation via flowering time modulation were advantageous in founder populations inhabiting pioneer habitats [32].

### Altered FCA-like protein function from a structural framework

We investigated the inferred slightly deleterious nature of longer polyQ tracts by examining the hypothetical structure of FCA-like proteins. In addition to the conserved WW protein-protein interaction domain, FCA-like proteins have two conserved RNA recognition (RRM) domains [17]. Along with the FY factor that binds to the WW domain, FCA is a component of a 3'-end RNA processing complex [33]. Aside from its well-defined domains, FCA-like proteins are unlike any other known protein family. However, detailed homology analysis and structural modeling based on multiple complete sequences reveals important structural features (see Additional file 1).

In order to function in an autoregulatory RNA processing complex, it is clear that simultaneous binding of RNA, FCA and the FY protein is required for physiological effect [24,33]. As such, the variable ca. 300 AA long intervening sequence between the second RRM and WW must have a well defined, rather rigid structure (Additional file 1). No strong matches were found by threading programs for the complete segment, or for parts of it, but various beta-folds dominate among the weak matches. The first 100 residues, which differ between monocots and dicots, show normal compositional variability and are likely to fold into regular secondary and tertiary structure. Two potentially stable, pseudo-dimeric 38 AA segments (labeled 3 and 4 in Additional file 1) occur in this region. The following segment of ca. 200 residues is enriched in glutamines and prolines, and poor on charged residues. Moreover, glutamines and prolines occur scattered throughout the entire length of this segment, spaced by 4–8 residues. Similar features are also observed after the WW domain. In several regions, the number of Q residues varies (from 3 to 9) among otherwise similar sequences (Figure 1; see also Additional file 1).

What could this rigid, Q-rich structure be like? One possibility is that a left-handed beta-helix would form. In these large structures of least 200 residues, beta strands and turns alternate to form a macrohelix that can be 50 Å long. For example, beta-helices are suggested to form in the long polyQ tracts of human disease-causing proteins past the critical value of Q_37 _[24]. We hypothesize a long beta helix covering the latter half of the RRM-WW linker and extending some 65 AA after the WW domain. The glutamines would favor beta-strand formation, and the prolines, the requisite beta-turns to form the tertiary helical structure [34]. The WW domain would fold separately as a loop structure, as is seen, for example, in the beta helical structure of penicillin dextranase (Protein Data Bank ID 1ogo.pdb). Similarly, the very C-terminal, non-repetitious 20 residues of FCA-like proteins should also arrange into a normal irregular fold and participate in ABA binding [18]. It could well be that the N-terminal RRM domains and the C-terminal WW domain come close to each other in three-dimensions, since beta sheets (present in the RRMs and beta helices) bind well to the sides of other beta sheets [35].

A structural/functional problem actively studied in relationship with Q-rich proteins is fibril formation, found in several neurogenerative diseases [24,35]. The longer the Q expansion, the more severe the effect [24]. In a beta-helix type of structure, long Q-stretches in FCA-like proteins could form an extra strand that would easily fit into the general fold. As a result, mutual orientation of the loops would change by about 120 degrees, and possible interactions between structural elements before and after the Q-repeat would be eliminated. In the mints, only Q-expansions up to 17 are observed, and whereas these would not be long enough to nucleate new structures, they would be sufficient to render the known functional domains of the FCA protein more mobile [cf. [36]], lessening the formation of functionally productive 3 end-processing complexes. As such, polyQ expansions could retard *FCA *homolog autoregulation and have deleterious physiological (and phenotypic) effects while not being long enough to permanently hinder folding of the functional structure.

## Conclusion

The Hawaiian mint FCA-like system suggests the possibility that polyQ variation, as readily measured over a relatively short geological time sequence, contributed to morphological change and participated in incipient speciation. Paradoxically, these effects may have "taken advantage" of developmental pleiotropy by way of natural selection on genetic variation causing slightly deleterious protein function. This discovery supports suggestions that repetitive AA motifs might provide a general mechanism for driving morphological evolution [10], and that variation at such motifs might permit rapid tuning to environmental change [37-39]. Furthermore, our finding of substantial polyQ variation in FCA-like proteins across plants suggests the possibility that other species may modulate flowering time and simultaneously undergo morphological evolution via selection on polyQ repeat polymorphism.

Of great importance, however, is that the central hypothesis of this study must survive functional testing. This could be accomplished by heterologous or homologous transformation experiments with *fca *null Arabidopsis plants, the former by incorporating different mint alleles, the latter by inserting engineered *FCA *constructs with CAG repeats of increasing lengths. Furthermore, if the FCA/FY interaction does indeed become less stable with increasing polyglutamine length, then changes in alternative splicing of mint *FCA*-like RNA [22] might be detectable *in vivo*.

## Methods

### Database survey of FCA homologs

The organismic distribution of the polyQ tract was investigated by surveying databases for *FCA *homologs using sequential TBLASTN [40] searches, moving outwards from initial *Stenogyne *(EU005232), rice (AAW62371), and *Arabidopsis *(AAW38964) searches. Query sequences included the WW domain, some of the RRM-WW spacer, and parts of the C-terminus. Using this methodology, the sequences shown in Figure 1 and Additional file 1 were recovered, along with many others, including potato (*Solanum*), CV496389; tobacco (*Nicotiana*), EB428208; cotton (*Gossypium*), CO075196; peach (*Prunus*), DY654198; soybean (*Glycine*), BU083978; watermelon (*Citrullus*), DV737172; columbine (*Aquilegia*), DR916138; water lily (*Nuphar*), DT591009; maize (*Zea*), DY398660; loblolly pine (*Pinus*), CO165492; white spruce (*Picea*), DV987123; and moss (*Physcomitrella*), BJ590264.

### Plant material and DNA extraction

Plant materials were in most cases obtained from herbarium specimens. In some cases, fresh material, further dried in silica gel, was obtained during field work. Included in the study were a total of 44 Hawaiian endemic mint taxa and 5 putative hybrids (*N *= 92). Also included were a total of 44 *Stachys *species (*N *= 53) from throughout the geographic range of the genus. Taxon, voucher, and collection locality information is provided in Additional file 2. Genomic DNA from individual accessions was extracted either as described in [14] or using the DNeasy Plant Mini kit following the manufacturer's instructions (Qiagen Inc., Valencia, California, USA).

### SSR amplification and scoring

Simple sequence repeat (SSR) primers were identified using the free online tool SSR Primer [41] as described by [16]. Using homologous genomic DNA from *Stenogyne rugosa*, PCR amplifications were optimized by testing different PCR reagents and annealing temperatures. The following protocol proved successful: 10 μL reaction volume using the AmpliTaq Gold DNA Polymerase kit (Applied Biosystems, Foster City, California, USA), 0.2 mmol/L of a dNTP blend, 1 μmol/L of each primer, and 1 μL genomic, unquantified DNA, with a PCR touch-down protocol: 1) initial denaturation 95°C 10 min, 2) 10 cycles of 95°C 1 min, 60°C 1 min, decreasing annealing temperature 1°C/cycle, 72°C 1 min 30 sec, 3) 35 cycles of 95°C 1 min, 50°C 1 min, 72°C 1 min 30 sec, and 4) a final extension 72°C 10 min. Analysis of SSR variation was accomplished using a fluorescently labeled forward primer, size standard ROX500, and an ABI 3100 Genetic Analyzer (Applied Biosystems). Amplification profiles were scored using the GeneMapper Software v3.7 (Applied Biosystems).

To confirm the presence of CAG repeats and to determine the corresponding numbers of repeats to allele lengths, selected accessions of Hawaiian mints were analyzed with direct sequencing. Two homozygous accessions (*Stenogyne cranwelliae *1 and *Phyllostegia hispida*) were included, permitting a precise determination. PCR products were purified using 8 μL 10× diluted exoSAP-IT (USB Corporation) per reaction. Cycle sequencing, using the same primers as in the PCR reaction, was performed in 10 μL reactions using 2 μL BigDye Terminator Cycle Sequencing Ready Reaction Kit (Applied Biosystems), 10 pmol primer, and 3 μL cleaned PCR product. Sequencing products were purified with ethanol precipitation and analyzed using an ABI 3100 Genetic Analyzer (Applied Biosystems). Forward and reverse sequences were edited and aligned using Sequencher ver. 4.1.4 (GeneCodes, Ann Arbor, Michigan, USA).

### Analyses of SSR variation

Frequency distributions of alleles and statistical tests were calculated using the software SPSS v. 13.0 (SPSS Inc.). Frequency distributions were calculated for all data together and for subsamples from the four islands Hawai'i, Maui Nui, O'ahu, and Kaua'i. Maui Nui represents a single landmass now separated into the islands Kaho'olawe, Lana'i, Maui, and Moloka'i. Length differences among pooled alleles for these four populations were investigated using Kruskal-Wallis and one-way ANOVA tests. Since the Levene Test of Homogeneity of Variances was significant, the post hoc Tamhane's T2 test with ANOVA was performed (equal variances not assumed). 

### Curve fitting of SSR/morphological relationships

Morphological data were scored using information from [15] or from our own observations of available herbarium material when information was not recorded in this reference (*P. kaalaensis*, *P. renovans*, *P. waimeae*, and *S. cranwelliae*, the latter two taxa for nutlet size only). Measurements from the following morphological variables were scored: nutlet size, length of corolla lower and upper lips, number of flowers per verticillaster, corolla tube length, corolla size (estimated as a multiple of corolla (i) upper and (ii) lower lips, and (iii) tube lengths), length of pedicels and calyces, and leaf area (length × width). Hawaiian mint flowers are usually arranged in small, compact, axillary cymes, forming verticillate arrangements at each node or sometimes racemose inflorescences. The corollas are strongly zygomorphic and bilabiate and the fruits usually consist of four nutlets. For each morphological variable the relationship between allele length means (independent variable) and average values of the morphological variable (dependent variable) was investigated by linear, quadratic, and exponential curve fitting in SPSS for the following different groups of data points: all Hawaiian mint accessions, *Phyllostegia *accessions only, *Stenogyne *accessions only, and accessions from the four islands Hawai'i, Maui Nui, O'ahu, and Kaua'i, respectively. Since linear regressions gave the best fits in almost all cases, only these are reported here. Following common practice, no adjustments for multiple tests were made since (i) there are biological explanations for the null hypotheses to be rejected, and (ii) the results are meant to be exploratory, requiring further experimental confirmation.

### FCA structural analysis

Several threading programs were used to search for structural elements in FCA-like proteins [42-44]. Alignments were constructed using CLUSTALW [45], followed by hand adjustments. CHARMM C30B1 [46] was used for structural modeling of representative beta helices in the Protein Data Bank.

## Authors' contributions

CL and VAA designed the research. CL performed the research. CL, LL, and VAA analyzed the data and wrote the paper. All authors read and approved the final manuscript.

## Supplementary Material

Additional file 1 The experimentally known RNA recognition (RRM) domains and the WW domain are shown in boxes, with probable secondary structure marked (arrows for beta strands, cylinders for alpha helices). The hypothesized beta helix between the second RRM and the WW domain is marked with a thick green line under the alignment. Representative Q expansions are marked with asterisks, and the one analyzed here is boxed. Other boxes with numbers stand for likely beta domains. Segments 1a,b,c and 2a,b,c show weak mutual similarity, which is highlighted by the fact that the *Lolium perenne *sequence aligns best as 1a+1b+2c, as shown. However depending on the sequence set used, *Lolium *FCA can also align as 1a+1b+1c. A similar pseudo-dimeric structure is likely to exist for boxed domains 3 and 4. Sequences shown in the alignment: *T. aestivum *(*Triticum*), AAP84419 and AAP84418; *L. perenne *(*Lolium*), AAT72460; *O. sativa *(*Oryza*), AAW62371; *H. vulgare *(*Hordeum*), AAF97846; *S. officinarum *(*Saccharum*), CA085029; *A. thaliana *(*Arabidopsis*), AAW38964; *B. napus *(*Brassica*), AAL61622; *P. sativum *(*Pisum*), AAX20016; *M. truncatula *(*Medicago*), ABE82791; *Z. elegans *(*Zinnia*), AU291241; and *S. rugosa *(*Stenogyne*), EU005232. Click here for file

Additional file 2 Species, voucher, locality information and FCA SSR genotype for the individual accessions used in this study. Click here for file
