# Supplementary figures and images for: Polyglutamine variation in a flowering time protein correlates with island age in a Hawaiian plant radiation
Source: BMC Evol Biol. 2007 Jul 2;7:105. doi: 10.1186/1471-2148-7-105 (PMC1939987; doi:10.1186/1471-2148-7-105)

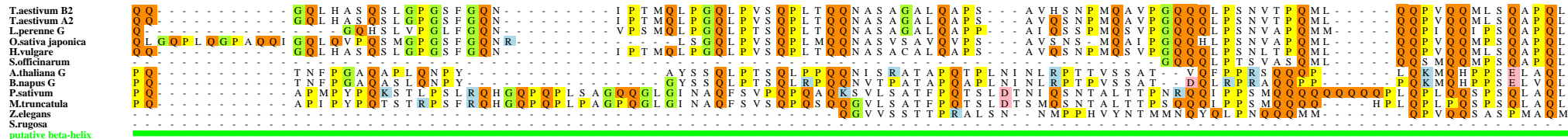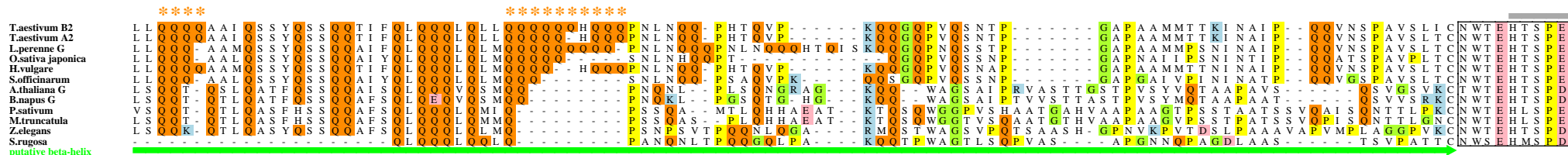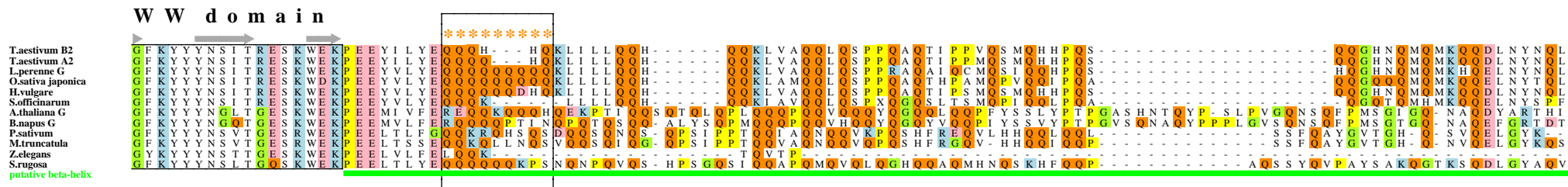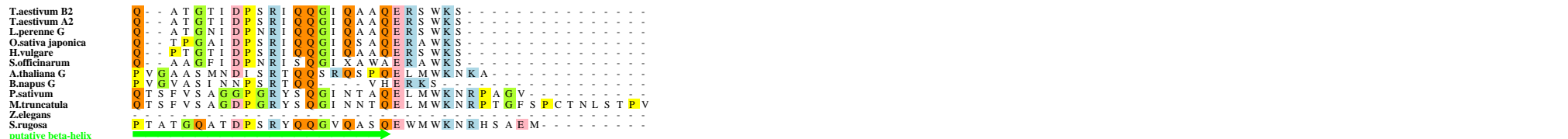

Supplement: Additional file 1 — The experimentally known RNA recognition (RRM) domains and the WW domain are shown in boxes, with probable secondary structure marked (arrows for beta strands, cylinders for alpha helices). The hypothesized beta helix between the second RRM and the WW domain is marked with a thick green line under the alignment. Representative Q expansions are marked with asterisks, and the one analyzed here is boxed. Other boxes with numbers stand for likely beta domains. Segments 1a,b,c and 2a,b,c show weak mutual similarity, which is highlighted by the fact that the Lolium perenne sequence aligns best as 1a+1b+2c, as shown. However depending on the sequence set used, Lolium FCA can also align as 1a+1b+1c. A similar pseudo-dimeric structure is likely to exist for boxed domains 3 and 4. Sequences shown in the alignment: T. aestivum (Triticum), AAP84419 and AAP84418; L. perenne (Lolium), AAT72460; O. sativa (Oryza), AAW62371; H. vulgare (Hordeum), AAF97846; S. officinarum (Saccharum), CA085029; A. thaliana (Arabidopsis), AAW38964; B. napus (Brassica), AAL61622; P. sativum (Pisum), AAX20016; M. truncatula (Medicago), ABE82791; Z. elegans (Zinnia), AU291241; and S. rugosa (Stenogyne), EU005232. [file 1471-2148-7-105-S1.pdf]
