# Supplementary material for: Polyglutamine variation in a flowering time protein correlates with island age in a Hawaiian plant radiation
Source: BMC Evol Biol. 2007 Jul 2;7:105. doi: 10.1186/1471-2148-7-105 (PMC1939987; doi:10.1186/1471-2148-7-105)
Supplement: Additional file 2 — Species, voucher, locality information and FCA SSR genotype for the individual accessions used in this study. [file 1471-2148-7-105-S2.doc]

**Additional file 2**

**Species, voucher, locality information and *FCA* SSR genotype for the individual accessions used in this study.**

| **Taxon** | **Voucher infomation^a^** | **Collection**  **locality** | **Genotype (allele length)** |
| --- | --- | --- | --- |
| *Haplostachys haplostachya* | C. Lindqvist et al. HI03-33 | Hawai’i | 93,99,102 |
| *Phyllostegia ambigua* | 1. R. Hobdy 3023 (BISH) | East Maui | 117,120 |
|  | 2. G. Clarke 688 (BISH) | Hawai’i | 99,102,105,108 |
|  | 3. C. Lindqvist et al. HI03-59 | Hawai’i | 105,108,111,114,117,120,  123,126,129,132,135 |
| *P. bracteata* | B.H. Gagne s.n., 1981 (BISH) | Maui | 93,99,102 |
| *P. brevidens* | 1. J. Griffin s.n., 1985 (BISH) | Hawai’i | 99,102,108,111 |
|  | 2. K. Wood 3200 (BISH) | East Maui | 99,102 |
| *P. electra* | K. Wood 2967 (BISH) | Kaua’i | 90,93,96,99,102 |
| *P.* cf*. electra* | 1. J.J. Fay 156 (NY) | Kaua’i | 99,102 |
|  | 2. C. Lindqvist et al. HI03-34 | Kaua’i | 99,105,108 |
| *P. floribunda* | 1. J.D. Jacobi 1326 (BISH) | Hawai’i | 90,96,99 |
|  | 2. C. Lindqvist et al. HI03-63 | Hawai’i | 96,99 |
| *P. glabra* var. *glabra* | 1. W.L. Wagner 5761 (BISH) | Moloka’i | 99,102 |
|  | 2. J. Obata s.n., 1990 (BISH) | O’ahu | 99,102 |
| *P. glabra* x *grandiflora* | J. Lau 3538 (BISH) | O’ahu | 99,102 |
| *P. grandiflora* | P. Welton 801 (BISH) | O’ahu | 99,102 |
| *P. haliakalae* | D. Herbst 4048 (BISH) | Lana’i | 102,105 |
| *P. hirsuta* | J. Obata s.n., 1993 (BISH) | O’ahu | 93,99,102 |
| *P. hispida* | L. Stemmermann 3973 (BISH) | Moloka’i | 99 |
| *P. kaalaensis* | 1. J. Obata & R. Robichaux 407 (BISH) | O’ahu | 105,108 |
|  | 2. S. Perlman 6117 (BISH) | O’ahu | 90,96,99 |
|  | 3. W. Takeuchi & Paquin 3440 (BISH) | O’ahu | 90,96,99 |
|  | 4. W. Takeuchi 941 (BISH) | O’ahu | 96,99 |
| *P. kahiliensis* | W.L. Wagner 5217 (BISH) | Kaua’i | 93,99,102 |
| *P. knudsenii* | K. Wood 2583 (BISH) | Kaua’i | 81,90 |
| *P. lantanoides* | J. Obata 86-624 (BISH) | O’ahu | 84,93 |
| *P. macrophylla* | 1. F.R. Warshauer 2862 (BISH) | East Maui | 99,105,108 |
|  | 2. C. Lindqvist et al. HI03-54 | Hawai’i | 120,123,126 |
| *P. mannii* | F.R. Warshauer 2418 (BISH) | Moloka’i | 111,114,117 |
| *P. parviflora* | J. Obata s.n., 1990 (BISH) | O’ahu | 93,99,102 |
| *P. racemosa* | 1. C. Lindqvist et al. HI03-57 | Hawai’i | 96,102,105 |
|  | 2. F.R. Warshauer 1447 (BISH) | Hawai’i | 102,105 |
| *P. renovans* | S. Perlman 10830 (BISH) | Kaua’i | 99,102 |
| *P.* cf*. renovans* | S. Perlman 13256 (BISH) | Kaua’i | 93,99,102 |
| *P. rockii* | C.N. Forbes 199 (BISH) | East Maui | 99,102 |
| *P. stachyoides* | 1. F.R. Warshauer 1856 (BISH) | Hawai’i | 96,102,105 |
|  | 2. J.S. Meidell 111 (BISH) | West Maui | 105,108 |
|  | 3. K. Wood 6280 (BISH) | Moloka’i | 99,105,108 |
| *P. velutina* | 1. C. Lindqvist et al. HI03-61 | Hawai’i | 111,114 |
|  | 2. J. Griffin s.n., 1992 (BISH) | Hawai’i | 105,108,111,114 |
| *P. vestita* | 1. St. John 22360 (NY) | Hawai’i | 96,111,114 |
|  | 2. C. Lindqvist et al. HI03-62 | Hawai’i | 105,108,111,114 |
| *P. waimeae* | C. Lindqvist et al. HI03-27 | Kaua’i | 93,99,102 |
| *P. warshaueri* | 1. C. Lindqvist et al. HI03-30 | Hawai’i | 93,99,102,105,108 |
|  | 2. S. Perlman 14185 (BISH) | Hawai’i | 99,102 |
| *P. wawrana* | S. Perlman 13690 (NY) | Kaua’i | 99,102 |
| *Stenogyne angustifolia* | R. Hobdy 2451 (BISH) | Hawai’i | 99,102,105,108 |
| *S.* cf*. angustifolia* | C. Lindqvist & V.A. Albert 32 (NY) | Hawai’i | 99,102 |
| *S. bifida* | 1. C. Lindqvist et al. HI03-32 | Moloka’i | 93,99,102 |
|  | 2. F.R. Warshauer 2377 (BISH) | Moloka’i | 93,99,102 |
|  | 3. F.R. Warshauer 3028 (BISH) | Moloka’i | 93,99,102 |
|  | 4. K. Wood 6284 (BISH) | Moloka’i | 99,102 |
| *S. calaminthoides* | 1. C. Lindqvist et al. HI03-04 | Hawai’i | 93,99,102 |
|  | 2. C. Lindqvist & V.A. Albert 349 (NY) | Hawai’i | 102,105,108,111 |
|  | 3. C. Lindqvist & V.A. Albert 42 (NY) | Hawai’i | 102,111 |
|  | 4. C. Lindqvist & V.A. Albert 82 (NY) | Hawai’i | 99,102,108,111 |
| *S. calycosa* | R. Hobdy 2553 (BISH) | East Maui | 93,99,102 |
| *S. campanulata* | 1. C. Lindqvist et al. HI03-36 | Kaua’i | 84,87,93,96 |
|  | 2. K. Wood 1790 (BISH) | Kaua’i | 87,93,96 |
| *S. cranwelliae* | 1. C. Lindqvist et al. HI03-64 | Hawai’i | 93 |
|  | 2. J. Davis 945 (BISH) | Hawai’i | 105,108,114,117 |
| *S. kaalae* | K. Nagata 1617 (NY) | O’ahu | 93,99,102 |
| *S. kamehamehae* | 1. P.K. Higashino 9461 (BISH) | Moloka’i | 93,99,102 |
|  | 2. W.L. Wagner 5888 (BISH) | East Maui | 99,102 |
| *S. kanehoana* | 1. J. Obata 331 (BISH) | O’ahu | 99,102,108,111 |
|  | 2. J. Obata 356 (BISH) | O’ahu | 108,111 |
| *S. kealiae* | C. Lindqvist et al. HI03-31 | Kaua’i | 87,93,96 |
| *S. macrantha* | 1. W. Mull & M. Mull s.n., 1980 (BISH) | Hawai’i | 102,105,111,114 |
|  | 2. C. Lindqvist et al. HI03-60 | Hawai’i | 96,102,105 |
|  | 3. C. Lindqvist et al. HI03-68 | Hawai’i | 102,105 |
| *S. microphylla* | 1. F.R. Warshauer 2682 (BISH) | Maui | 96,99,102,105 |
|  | 2. C. Lindqvist & V.A. Albert 178 (NY) | Hawai’i | 99,102,105,108 |
|  | 3. C. Lindqvist & V.A. Albert 85 (NY) | Hawai’i | 93,99,102 |

| *S.* cf*. microphylla* x *rugosa* | 1. C. Lindqvist & V.A. Albert 38 (NY) | Hawai’i | 99,105,108 |
| --- | --- | --- | --- |
|  | 2. C. Lindqvist & V.A. Albert 107 (NY) | Hawai’i | 93,99,102 |
|  | 3. C. Lindqvist & V.A. Albert 288 (NY) | Hawai’i | 102 |
|  | 4. C. Lindqvist & V.A. Albert 306 (NY) | Hawai’i | 99,102 |
| *S. purpurea* | 1. C. Lindqvist et al. HI03-40 | Kaua’i | 108,111 |
|  | 2. K. Wood 1772 (BISH) | Kaua’i | 87,93,96 |
|  | 3. C. Lindqvist & V.A. Albert 68 (NY) | Kaua’i | 108,111 |
| *S. rotundifolia* | F.R. Warshauer 2545 (BISH) | Maui | 99,102 |
| *S. rugosa* | 1. C. Lindqvist et al. HI03-65 | Hawai’i | 105,108,111,114 |
|  | 2. C. Lindqvist et al. HI03-73 | Hawai’i | 93,99,102 |
|  | 3. C. Lindqvist & V.A. Albert 148 (NY) | Hawai’i | 93,99,102 |
|  | 4. C. Lindqvist & V.A. Albert 63 (NY) | Hawai’i | 99,102,108,111 |
|  | 5. B.H. Gagne s.n., 1975 (BISH) | Maui | 102,105 |
| *S. scrophularioides* | 1. W.L. Wagner 5954 (BISH) | Hawai’i | 99,102,105 |
|  | 2. C. Lindqvist et al. HI03-58 | Hawai’i | 102,108,111 |
| *S. sessilis* | 1. C. Lindqvist et al. HI03-67 | Hawai’i | 96,99,102,105 |
|  | 2. O. Degener 33639 (NY) | Hawai’i | 99,102 |
|  | 3. S.G. Weller 821 (BISH) | Hawai’i | 99,102 |
|  | 4. S. Perlman 15398 (BISH) | Maui | 99,102 |
| *Stachys aculeolata* | Y.B. Harvey et al. 7 (C) | Kenya | 99,105 |
| *Sta. aethiopica* | B. Pettersson 2146 (UPS) | Mozambique | 90,96,99 |
| *Sta. affinis* | C. Lindqvist & V.A. Albert 359 (UNA) | cult., E Asia | 96,99,102,105,108,111 |
| *Sta. agraria* | G. Nesom 6113 (TEX) | Mexico | 96 |
| *Sta. albens* | G. Baird 1630 (RM) | Utah | 87,93,96 |
| *Sta. albotomentosa* | H. Rubio 1984 (TEX) | Mexico | 87,93,96,99,105,108 |
| *Sta. alpigena* | O. Ryding 2133 (UPS) | Ethiopia | 102,105 |
| *Sta. ampla* | H. Hapeman s.n., 1938 (UPS) | S Dakota | 87,96 |
| *Sta. argillicola* | I. Friis et al. 3104 (C) | Ethiopia | 93,99,102 |
| *Sta. arvensis* | 1. N. Lundqvist 8157 (UPS) | Tenerife | 93 |
|  | 2. O. Ryding 2394 (C) | Tenerife | 93 |
| *Sta. aspera* | J.B. Nelson 1326 (UNA) | Florida | 93,99,102,105,108 |
| *Sta. baicalensis* | H. Takahashi 2950 (C) | Japan | 87,93,96,99,102 |
| *Sta. boraginoides* | P. Cruz M. s.n., 1982 (LL) | Mexico | 93,96 |
| *Sta. bullata* | M.R. Crosby & N. Morin 14355 (RM) | California | 87,96,99,102 |
| *Sta. byzantina* | C. Lindqvist & V.A. Albert 356 (UNA) | cult., Greece | 87,93,96 |
| *Sta. chamissonis* | 1. H.N. & A.L. Moldenke 32097 (LL) | Oregon | 93,99,102 |
|  | 2. S. Jennings 218 (UTC) | Washington | 87,93,96 |
| *Sta. chrysantha* | Bergmeier 95-317 (C) | Greece | 93,99,102,111 |
| *Sta. coccinea* | 1. J. Ricketson & L. Raechal 4274 (TEX) | New Mexico | 87,90,93,96,99 |
|  | 2. C. Lindqvist & V.A. Albert 355 | cult., NYBG 911/97A | 87,93,96,99,102 |
| *Sta. cretica* | A. Strid et al. 42603 (C) | Greece | 93,96,99,102,105 |
| *Sta. drummondii* | 1. B. Ertter 5530 (UTC) | Texas | 87,90,93,96,102 |
|  | 2. C.J. Ferguson 72 (TEX) | Texas | 87,93,96,99,102,105 |
| *Sta. eriantha* | A. McDonald & G. Nesom 2495 (TEX) | Mexico | 96 |
| *Sta. floridana* | M. Kortright 102 (UNA) | Alabama | 90,96,99 |
| *Sta. grahamii* | G.B. Hinton et al. 24399 (TEX) | Mexico | 96 |
| *Sta. lamioides* | E. Asplund 17092 (US) | Ecuador | 87,93,96 |
| *Sta. langmaniae* | 1. J.A. Villarreal 5084 (TEX) | Mexico | 96,99,102,105 |
|  | 2. McDonald 1620 (TEX) | Mexico | 99 |
| *Sta. latidens* | J.A. Churchill 83034 (RM) | N Carolina | 87,93,96 |
| *Sta. lavandulaefolia* | J.A. Andersen & A.G. Jensen 7032 (C) | Iran | 90,96,99 |
| *Sta. lindenii* | 1. P. Tenorio L. 11084 (TEX) | Mexico | 87,93,96,102 |
|  | 2. R. Torres C. 4602 (TEX) | Mexico | 87,90,93,96,99,102 |
| *Sta. nepetifolia* | I. Diaz V. 44 (TEX) | Mexico | 87,93,96,99 |
| *Sta. nuttallii* | J.B. Nelson 14361 (UNA) | S Carolina | 87,93,96 |
| *Sta. pacifica* | 1. G. Flores F. 2344 (TEX) | Mexico | 93,96,99,102,105 |
|  | 2. M. Fishbein et al. 2133 (TEX) | Mexico | 93,96,99,102 |
| *Sta. pilosa* | 1. G.E. Larson 10569 (RM) | S Dakota | 87,93,96 |
|  | 2. T. Cramer 1909 (RM) | Wyoming | 87,93,96 |
| *Sta. quercetorum* | 1. G.K. Helmkamp 2153 (UTC) | California | 87,93,96 |
|  | 2. R.F. Thorne 61281 (RM) | California | 99,102,105,108 |
| *Sta. radicans* | D.E. Breedlove 51924 (TEX) | Mexico | 90,96,102 |
| *Sta. rigida* | H.N. Moldenke et al. 32116 (LL) | Oregon | 96,99,102,105,108 |
| *Sta. rivularis* | A. Tiehm 12609 (UTC) | Nevada | 87,93,96,99,102 |
| *Sta. rothrockii* | E. Neese et al. 15716 (RM) | Utah | 93,99,102 |
| *Sta. rotundifolia* | D.E. Breedlove 55575 (TEX) | Mexico | 90,96,96,99 |
| *Sta. setifera* | J.S. Andersen & I.C. Petersen 115 (C) | Iran | 87,96 |
| *Sta. swainsonii* | A. Strid et al. 39692 (C) | Greece | 93,99,102 |
| *Sta. sylvatica* | C. Lindqvist & V.A. Albert 358 (UNA) | cult., Eurasia | 90,93,96,99 |
| *Sta. tenuifolia* | R.L. McGregor 31847 (RM) | Kansas | 87,93,96 |
| *Sta. torresii* | A. McDonald 2927 (TEX) | Mexico | 87,93,96,99 |
| *Sta. vulnerabilis* | G.B. Hinton et al. 24774 (TEX) | Mexico | 87,93,96,99,102 |

^a^Herbaria abbreviations in parentheses follow Holmgren et al. (1990).

**Supplementary reference**

Holmgren PK, Holmgren NH, Barrett LC: *Index Herbariorum. Part I. The herbaria of the world*. Bronx, NY: New York Botanical Garden Press; 1990.
